# Methylene blue in sepsis and septic shock: a systematic review and meta-analysis

**DOI:** 10.3389/fmed.2024.1366062

**Published:** 2024-04-18

**Authors:** Raquel Simões Ballarin, Taline Lazzarin, Leonardo Zornoff, Paula Schmidt Azevedo, Filipe Welson Leal Pereira, Suzana Erico Tanni, Marcos Ferreira Minicucci

**Affiliations:** Internal Medicine Department, Medical School, São Paulo State University (UNESP), Botucatu, Brazil

**Keywords:** sepsis, septic shock, methylene blue, meta-analysis, systematic review

## Abstract

**Background:**

Methylene blue is an interesting approach in reducing fluid overload and vasoactive drug administration in vasodilatory shock. The inhibition of guanylate cyclase induced by methylene blue infusion reduces nitric oxide production and improves vasoconstriction. This systematic review and meta-analysis aimed to assess the effects of methylene blue administration compared to placebo on the hemodynamic status and clinical outcomes in patients with sepsis and septic shock.

**Methods:**

The authors specifically included randomized controlled trials that compared the use of methylene blue with placebo in adult patients with sepsis and septic shock. The outcomes were length of intensive care unit stay, hemodynamic parameters [vasopressor use], and days on mechanical ventilation. We also evaluated the abnormal levels of methemoglobinemia. This systematic review and meta-analysis were recorded in PROSPERO with the ID CRD42023423470.

**Results:**

During the initial search, a total of 1,014 records were identified, out of which 393 were duplicates. Fourteen citations were selected for detailed reading, and three were selected for inclusion. The studies enrolled 141 patients, with 70 of them in the methylene blue group and 71 of them in the control group. Methylene blue treatment was associated with a lower length of intensive care unit stay (MD −1.58; 95%CI −2.97, −0.20; *I*^2^ = 25%; *p* = 0.03), decreased days on mechanical ventilation (MD −0.72; 95%CI −1.26, −0.17; *I*^2^ = 0%; *p* = 0.010), and a shorter time to vasopressor discontinuation (MD −31.49; 95%CI −46.02, −16.96; *I*^2^ = 0%; *p* < 0.0001). No association was found with methemoglobinemia.

**Conclusion:**

Administering methylene blue to patients with sepsis and septic shock leads to reduced time to vasopressor discontinuation, length of intensive care unit stay, and days on mechanical ventilation.

**Systematic review registration:**

https://www.crd.york.ac.uk/prospero/display_record.php?ID=CRD42023423470, CRD42023423470.

## Introduction

Sepsis is an inadequate host response to an infection, resulting in organ dysfunction (1). It involves immunological, hormonal, and circulatory abnormalities such as peripheral vasodilatation. This vasodilatation comprises multifactorial mechanisms that include cytokine stimulation of inducible nitric oxide synthase and activation of various intrinsic vasodilatory pathways. Additionally, there is a diminished responsiveness to vasopressors (2–4). Treatment is mainly based on optimizing circulating volume, administering broad-spectrum antibiotics, and utilizing vasopressors to maintain a suitable mean arterial pressure (MAP) and organ perfusion (5, 6).

Following the research conducted by Rivers et al. and the sepsis and septic shock treatment guided by early goal-directed therapy, there has been little progress in the therapeutic arsenal of this condition (4). The initial approach is fluid administration, but this is often insufficient to improve organ perfusion substantially. In such cases, vasopressor administration becomes necessary. Although norepinephrine is the first-choice vasoactive agent, there are some concerns with high doses that may cause potential adverse effects, such as increased myocardial oxygen consumption, tachyarrhythmias, myocardial ischemia, decreased regional blood flow, hyperglycemia, and hypercoagulability (7–10). Moreover, norepinephrine disrupts the immune response by reducing the production of proinflammatory mediators and reactive oxygen species while increasing the production of anti-inflammatory mediators. This compromise in host defense potentially contributes to the observed immunoparalysis in patients with septic shock (11). Furthermore, with prolonged exposure to high-dose catecholamines, adrenergic receptors experience downregulation and desensitization, and consequently, some patients may exhibit inadequate responsiveness (7, 12, 13).

In this context, strategies that prioritize decatecholaminization have been proposed for the management of patients with septic shock; however, as additional vasopressor agents, including vasopressin, terlipressin, and angiotensin-2, are similarly linked to an increased likelihood of tachyarrhythmias, organic ischemia, and immune dysfunction (9, 10), methylene blue (MB) has been explored as a potential alternative to reach the hemodynamic targets (14). MB acts by blocking the enzyme guanylate cyclase, reducing excessive nitric oxide production, and alleviating its vasorelaxant effect in vascular smooth muscle, restoring vascular tone and increasing blood pressure (10, 15, 16).

Although MB effectively increases vascular tone and arterial pressure, its affordability and widespread availability notwithstanding, the absence of randomized clinical trials poses challenges in evaluating its effectiveness in patients with sepsis (10). While several systematic reviews have previously been published on this subject matter, recently, a randomized controlled trial (RCT) with a larger sample was published (17). Hence, we conducted an updated systematic review and meta-analysis with the aim of assessing the impact of MB administration, in comparison to a placebo, on both hemodynamic status and clinical outcomes in patients with sepsis and septic shock.

## Methods

### Inclusion criteria

The protocol for this review was recorded in the Prospective International Register of Systematic Reviews (PROSPERO) with the ID CRD42023423470. We conducted our study following the Preferred Reporting Items for Systematic Reviews and Meta-Analyses (PRISMA) guidelines (18). The search strategy was based on the PICO (P: Patient; I: Intervention; C: Comparison; O: Outcome) methodology. The PICO framework is as follows: Patient—adult (more than 18 years old) patients with sepsis and septic shock (Sepsis or septic shock was defined according to the current consensus criteria at the time the studies were performed) (1, 19); Intervention—the use of MB; Comparison—the control group receiving standard treatment without MB; and Outcome—mortality rate, length of intensive care unit (ICU) stay, hemodynamic parameters [MAP and vasopressor use], and days on mechanical ventilation. We also evaluated the side-effect levels of methemoglobinemia (MHb). Only RCT designs were considered for inclusion.

### Search strategy

Two researchers independently searched three electronic databases, including MEDLINE, EMBASE, and the Cochrane Library, for studies assessing the MB role in sepsis or septic shock until May 2023. Language or period limitations were not applied during the search process. Our search terms were selected according to the following terms: “methylene blue,” “sepsis,” “septic shock,” and their respective synonyms. The complete search strategy for each database can be found in the Supplementary Table S1.

### Study selection and data extraction

The search results were entered into Rayyan software (20). After removing the duplicates, two reviewers (R.S.B. and T.L.) independently screened all titles and abstracts and selected full articles for review. Disagreements on study selection were settled by a third reviewer (M.F.M.). Two authors independently gathered data from all eligible trials, encompassing study design and methodology, patient characteristics, and descriptions of interventions. To perform statistical analysis, medians and interquartile ranges were transformed into means and standard deviations using an online calculator (21, 22).

### Risk of bias

Two researchers (R.S.B. and T.L.) independently evaluated the risk of bias (RoB) in the studies, and disagreements were resolved in consultation with the third researcher (M.F.M.). The Cochrane Risk of Bias (RoB) 2.0 tool (23) was used to determine the adequacy of the randomization process, deviations from the intended interventions, missing outcome data, measurement of the outcome, selection of the reported result, and the overall RoB for each study.

### Certainty of evidence assessment

We utilized the Grading of Recommendations, Assessment, Development, and Evaluations (GRADE) (24) system to evaluate the quality of evidence. This system assesses methodological limitations, inconsistency, imprecision, indirectness, and publication bias to categorize the quality of evidence as very low, low, moderate, or high. The summary of the table was created using GRADEpro GDT software.

### Synthesis of results and analysis

The statistical analyses were conducted using Review Manager 5.4.1, and a *p*-value of < 0.05 was defined as statistical significance. Dichotomous outcomes were reported as the risk ratio (RR) and continuous outcomes as the mean difference (MD), both with a 95% confidence interval (CI). The weight of each study was calculated using the inverse variance method, and a random-effects model was used to explore MB intervention, as this considers the heterogeneity between RCTs. The heterogeneity between RCTs was assessed using *I*^2^, with substantial heterogeneity identified as *I*^2^ > 50%. Due to the small sample size of RCTs, no subgroup analysis was performed.

## Results

**Figure 1:**
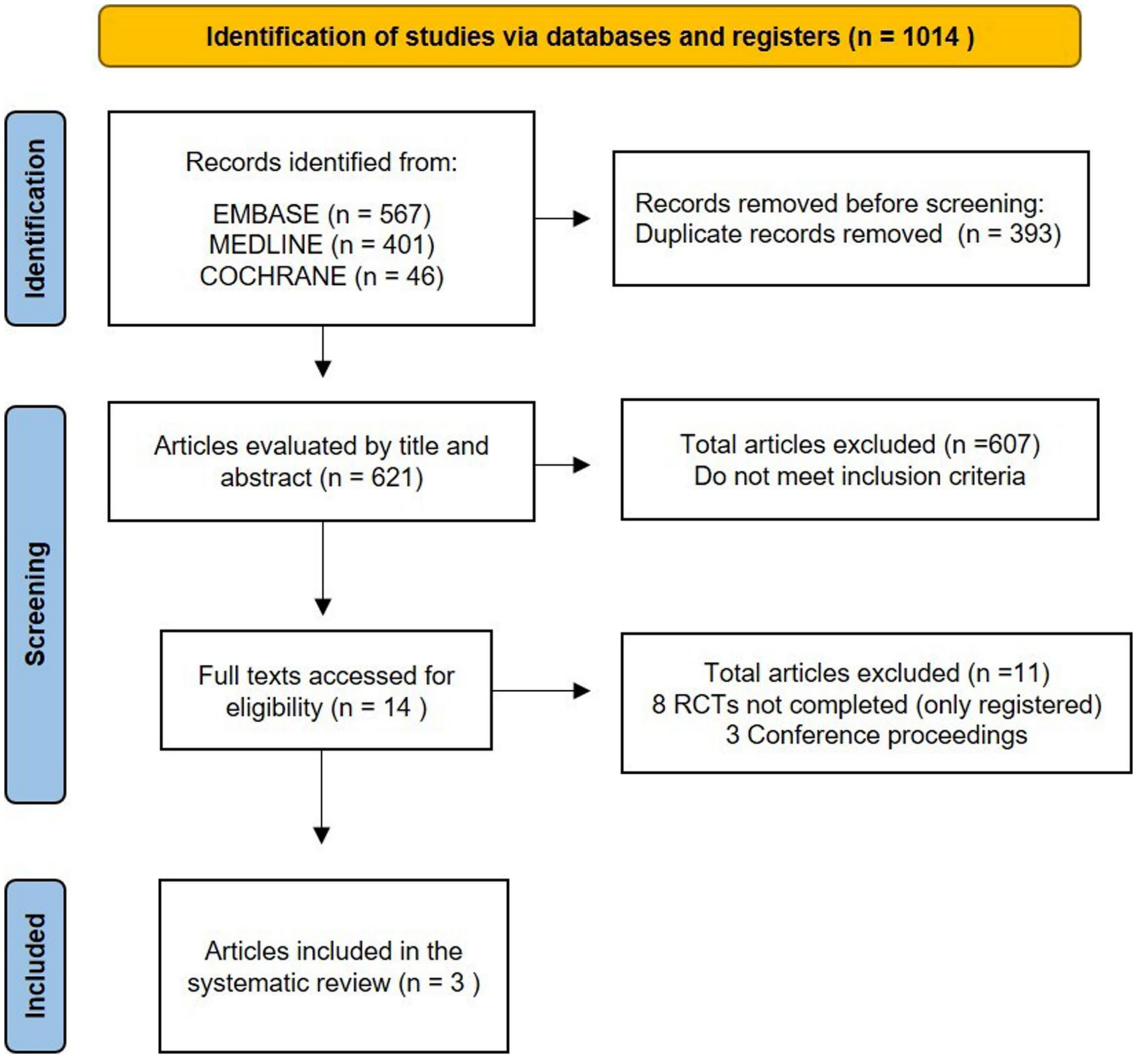
Flowchart of the article selection process.

We identified 1,014 records during the initial search, out of which 393 were duplicates. Thus, the total number of abstracts and titles screened was 621. It led to 607 records being excluded and 14 full texts being screened for eligibility. Out of these 14 studies, eight trials were excluded because they are still in progress (five of them with unclear status), and three were conference abstract publications (Figure 1; Supplementary Table S2). Therefore, three studies were included (Table 1) (17, 25, 26). All studies in the data synthesis were RCTs with two arms (MB and control groups), including 141 patients, with 70 of them in the MB group and 71 of them in the control group. Of these, 111 patients had septic shock and 30 had sepsis (severe sepsis, the terminology prevailing at the time, more specifically). One of them was a pilot study (26). Details of these trials and MB infusion are available in Table 1.

**Table 1:** Summary characteristics of the included studies.

Author, year	Language	No patients	Intervention (MB)	Outcomes	Results
Kirov, 2001	English	20 (10 control/10 MB)	2 mg/kg IV bolus followed by an infusion with a stepwise increase in the rate of 0.25, 0.5, 1, and 2 mg/kg/h maintained for 1 h each	Evaluate the effects of continuously infused MB on hemodynamics, gas exchange, and other organ function variables MAP, PAOP, HR, mean CVP, and PAP, cardiac output, Arterial and mixed venous blood gasses * MHB, SOFA score, duration of septic shock, vasopressor support, MV, hospital and ICU stays, number of organ dysfunctions at 24 h, details of sedation and fluid therapy from 0 to 24 h, and survival rate at day 28 †	↑ MAP * Counteracts myocardial depression * Maintains oxygen transport * ↓ Vasopressor requirements †
Memis, 2002	English	30 (15 control/15 MB)	0.5 mg/kg/h IV continuous infusion via a central venous catheter for 6 h	Plasma levels of cytokines* Change in CVP, MAP, HR, pH, PO2, PCO2, and SaO2, platelets, leukocytes, bilirubin, ALT and creatinine, MHB, duration of MV was recorded and mortality †	↑ MAP † ↑ MHB †
Ibarra-Estrada, 2023	English	91 (46 control/45 MB)	MB 100 mg in 500 mL of 0.9% sodium chloride solution over 6 h once daily for a total of three doses	Time to vasopressor discontinuation* Vasopressor-free days at 28 days, all-cause mortality at 28 days, serum lactate levels, days on MV, length of stay in ICU and hospital; change in serum creatinine, bilirubin, ALT/AST, PaO2/FIO2 ratio and ejection fraction after intervention †	↓ Vasopressor requirements † ↓ ICU and hospital length of stay † ↓ cumulative fluid balance †

MB, methylene blue; MAP, mean arterial pressure; HR, heart rate; PAOP, pulmonary artery occlusion pressure; MHB, methemoglobinemia; ICU, intensive care unit; CVP, central venous pressure; PAP, mean pulmonary arterial pressure; MV, mechanical ventilation; ALT, alanine aminotransferase; AST, aspartate aminotransferase.

^*^Primary outcomes.

^†^Secondary outcomes.

The evaluation of the bias risk was conducted using the Cochrane Collaboration tool (Supplementary Figure S1). No study scored a low RoB in all domains. However, all studies manifest some concerns about the domain selection of the reported results due to the absence of a pre-specified protocol before the analysis was performed.

**Figure 2:**
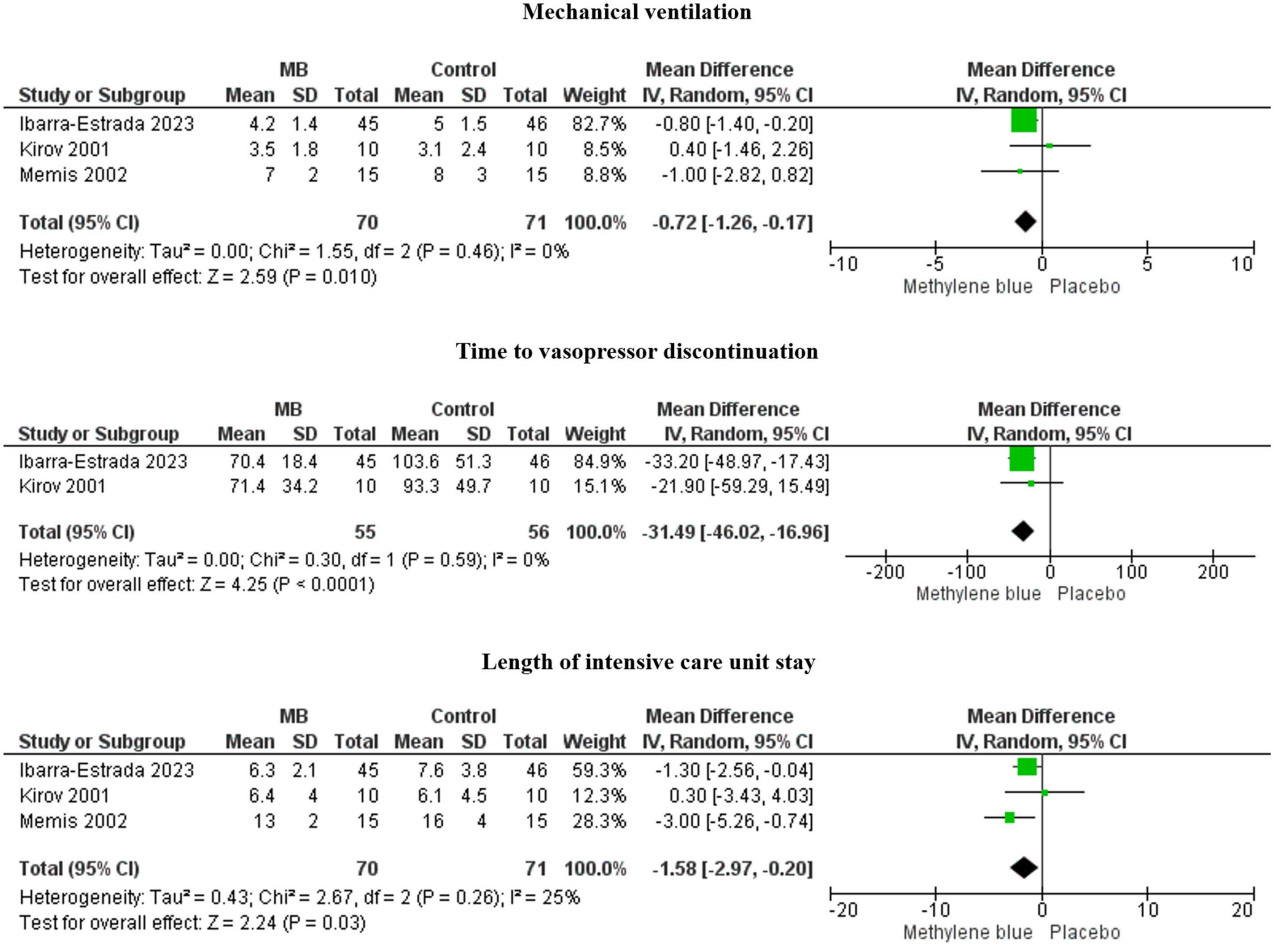
Forests plots of time to vasopressor discontinuation, length of ICU stay, and days on mechanical ventilation.

MB treatment was associated with decreased days on mechanical ventilation (MD, −0.72; 95%CI, −1.26 to −0.17; *I*^2^ = 0%; *p* = 0.010), a lower length of ICU stay (MD, −1.58; 95%CI, −2.97 to −0.20; *I*^2^ = 25%; *p* = 0.03), and less time to vasopressor discontinuation (MD, −31.49; 95%CI, −46.02 to −16.96; *I*^2^ = 0%; *p* < 0.0001) (Figure 2).

These three essential outcomes, with low heterogeneity, were statistically significant in the overall effect analysis.

As there is no consistent standardization among studies for assessing mortality [one study (25) used hospital mortality and others (17, 26) used 28-day mortality], and since there was difficulty in extracting data on MAP in one of the studies (17), we opted not to utilize this information to prevent potential bias.

Additionally, we performed a meta-analysis to evaluate the levels of MHb, the main side-effect event related to MB infusion. No statistical difference was found in MHb, including all trials that evaluated MB (MD, 0.85; CI95%, −0.44 to 2.15; *I*^2^ = 99%; *p* = 0.20) (Supplementary Figure S2).

We assessed the certainty of evidence using GRADE (24) and the funnel plot for each outcome (Supplementary Figure S3; Supplementary Table S3). Regarding vasopressor use, there was moderate certainty evidence that MB decreases the time to vasopressor discontinuation compared to usual care. There is low certainty evidence that MB reduces the length of ICU stay and days on mechanical ventilation due to inconsistency and imprecision. The evidence was downgraded because of variation in the estimated treatment effects, no optimal sample size information, a large CI, and heterogeneity.

## Discussion

This systematic review and meta-analysis evaluated the effect of MB in three RCTs. They found that MB treatment significantly reduced time to vasopressor discontinuation, days on mechanical ventilation, and length of ICU stay.

MB is an interesting approach in reducing fluid overload and vasoactive drug administration. The inhibition of guanylate cyclase induced by MB infusion reduces nitric oxide production and improves vasoconstriction. Based on this rationale, MB was administered in different scenarios, mainly in patients with vasodilatory shock. However, contradictory results were observed regarding improving systemic vascular resistance and mortality (27, 28). Moreover, MB was only administered to patients with sepsis and septic shock in the three selected RCTs for this study.

Kirov et al. (26) performed a randomized, controlled, open-label pilot study that randomized 20 patients with septic shock to receive either MB or isotonic saline adjunctive to sepsis conventional treatment. MB was administered through a bolus injection of 2 mg/kg, followed 2 h later by increasing rates of 0.25, 0.5, 1, and 2 mg/kg/h, sustained for 1 h each. Hemodynamic and organ function variables were evaluated continuously for 24 h, and the survival rate on day 28 was documented. The MB infusion had no adverse effects. There was also improvement in myocardial depression, oxygen delivery, and reduced vasoactive drug support (26).

Memis et al. (25) performed a prospective, randomized, double-anonymized, placebo-controlled study to evaluate the effect of MB infusion on plasma levels of cytokines in 30 patients with severe sepsis. Patients were randomly assigned to receive either an MB infusion of 0.5 mg/kg/h or a similar volume of isotonic saline for 6 h. Plasma concentrations of inflammatory biomarkers, hemodynamics, and biochemical parameters were evaluated at baseline, immediately after MB infusion, and at 24 and 48 h after MB infusion. In addition, the duration of mechanical ventilation and hospital survival were recorded. MB infusion did not change cytokine levels or outcomes in severe sepsis; however, it resulted in a transient increase in arterial pressure.

Ibarra-Estrada et al. (17) performed the largest RCT with MB in patients with septic shock. They randomized 91 patients with septic shock to receive an intravenous infusion of 500 mL of 0.9% sodium chloride solution with or without 100 mg of MB over 6 h once daily for three doses. The primary outcome was time to vasopressor discontinuation at 28 days, while secondary outcomes included vasopressor-free days at 28 days, duration of mechanical ventilation, length of stay in the ICU and the hospital, and mortality at 28 days. Administering methylene blue infusion within 24 h of initiating norepinephrine resulted in a shorter time to vasopressor discontinuation, increased vasopressor-free days, and a reduction in the length of stays in the ICU and the hospital without adverse effects.

Summarizing these data, MB infusion improved hemodynamic status, decreased length of ICU stay, and days on mechanical ventilation. In addition, there was no effect on MHb levels, suggesting that this intervention is safe. The different populations, methods, dosages, and timing of MB administration can explain divergent results in some studies. Early MB start, in the first 8 h of sepsis, and continuous infusion for a longer time, due to a half-life of 5–6 h, are probably more effective (12, 28). A recent cohort study demonstrated that the method of MB administration may impact its efficacy in patients experiencing shock, with a reduction in 28-day mortality observed in the group receiving MB through bolus injection followed by continuous infusion (29).

When interpreting the findings of this study, it is important to take into account several limitations. Despite including only RCTs, the study has a small sample size. Another practical limitation to be considered is the availability of MB in some European countries. In addition, the degree of certainty of most outcomes was considered low by the GRADE tool (24). Despite these limitations, our analysis adds information about a potential new approach for treating sepsis and septic shock patients. In addition, this meta-analysis could help physicians make decisions in clinical practice.

In this context, considering its established safety profile and affordability, MB can be viewed as a viable option for reducing the use of catecholamines. Importantly, other RCTs of MB administration in patients with sepsis and septic shock are needed to define the ideal dose, timing, duration of treatment, and the best subgroup of patient’s candidates for its use.

## Conclusion

Although more studies are necessary in the future, the findings of this meta-analysis suggest that MB could be a promising sparing vasopressor agent in patients with sepsis and septic shock.

## Data availability statement

The original contributions presented in the study are included in the article/Supplementary material, further inquiries can be directed to the corresponding author.

## Author contributions

RB: Conceptualization, Formal analysis, Methodology, Writing – original draft, Writing – review & editing. TL: Conceptualization, Formal analysis, Methodology, Writing – original draft, Writing – review & editing. LZ: Writing – original draft, Writing – review & editing. PA: Writing – original draft, Writing – review & editing. FP: Writing – original draft, Writing – review & editing. ST: Writing – original draft, Writing – review & editing. MFM: Conceptualization, Methodology, Supervision, Visualization, Writing – original draft, Writing – review & editing.
