# Supplementary material for: Methylene blue in sepsis and septic shock: a systematic review and meta-analysis
Source: Front Med (Lausanne). 2024 Apr 18;11:1366062. doi: 10.3389/fmed.2024.1366062 (PMC11063345; doi:10.3389/fmed.2024.1366062)
Supplement: Supplementary file 1 [file Data_Sheet_1.PDF]

Supplementary Figure 1. Risk of bias based on the Cochrane Collaboration tool

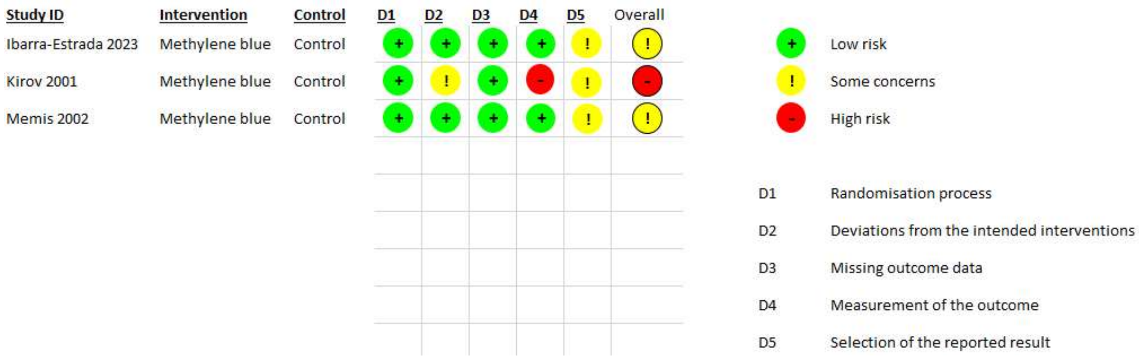

Supplementary Figure 2. Forrest plot of methemoglobinemia

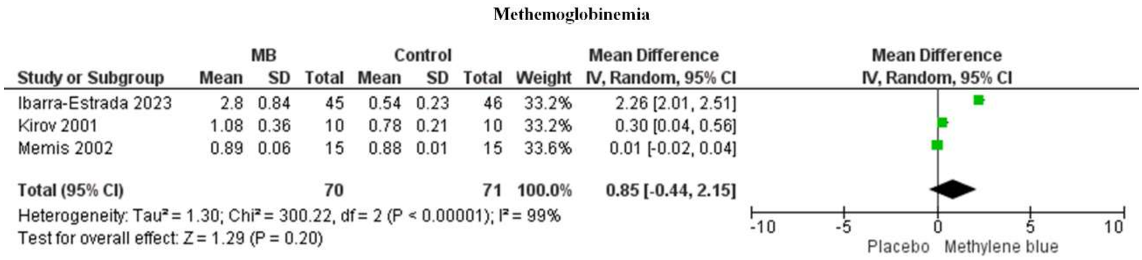

Supplementary Figure 3. Funnel plots

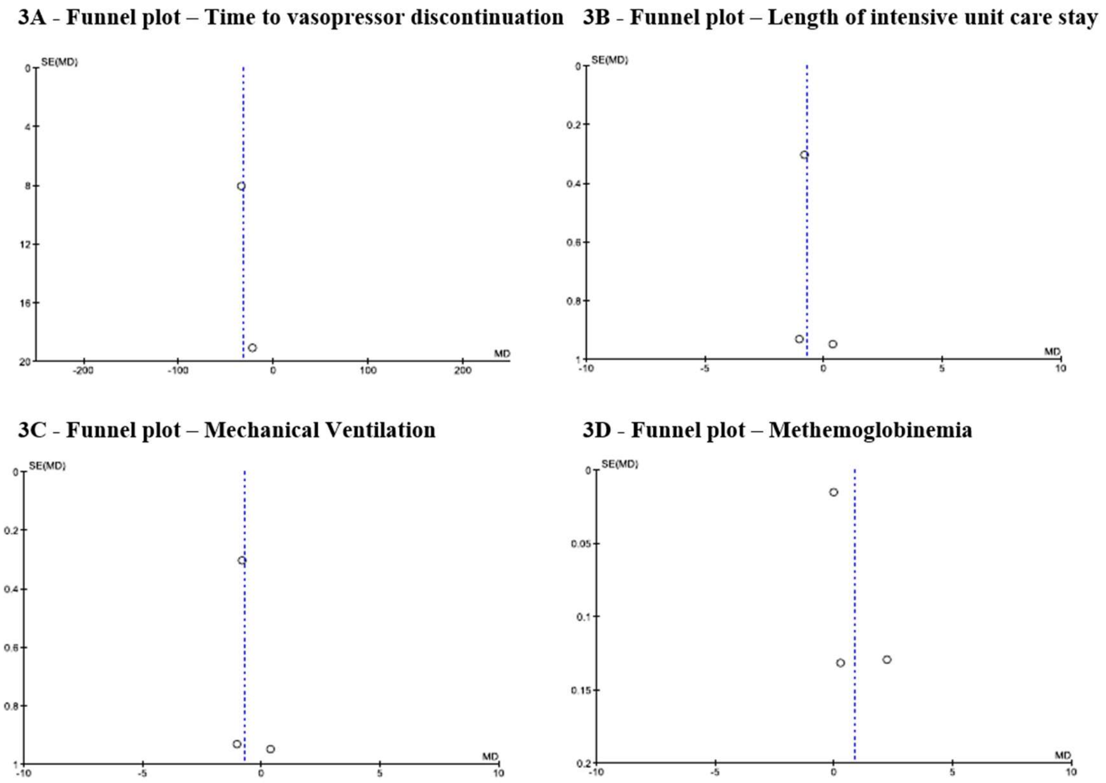

Supplementary Table 1. Electronic database search strategy

Database	Search strategy
MEDLINE	‘’Methylene blue’’ OR ‘’Methylthioninium Chloride’’ OR ‘’ Methylthionine Chloride’’ OR ‘’ Methylene Blue N’’ AND sepsis OR Septicemia OR Septicemias OR ‘’Severe Sepsis’’ OR ‘’Sepsis Syndrome’’ OR ‘’Sepsis Syndromes’’ OR ‘’Inflammatory Response Syndrome, Systemic’’ OR ‘’Septic Shock’’ OR ‘’Shock, Toxic’’ OR ‘’Toxic Shock Syndrome’’ OR ‘’Toxic Shock Syndromes’’ OR ‘’Shock, Endotoxic’’ OR ‘’Endotoxin Shock’’ OR ‘’Endotoxin Shocks’’
EMBASE	'methylene blue'/exp OR 'methylene blue' AND sepsis/exp OR sepsis OR septicemia/exp OR septicemia OR 'severe sepsis'/exp OR 'severe sepsis' OR 'systemic inflammatory response syndrome'/exp OR 'systemic inflammatory response syndrome' OR 'septic shock'/exp OR 'septic shock' OR 'toxic shock syndrome'/exp OR 'toxic shock syndrome' OR 'endotoxic shock'/exp OR 'endotoxic shock'
COCHRANE	‘’Methylene blue’’ OR ‘’Methylthioninium Chloride’’ OR ‘’ Methylthionine Chloride’’ OR ‘’ Methylene Blue N’’ AND sepsis OR Septicemia OR Septicemias OR ‘’Severe Sepsis’’ OR ‘’Sepsis Syndrome’’ OR ‘’Sepsis Syndromes’’ OR ‘’Inflammatory Response Syndrome, Systemic’’ OR ‘’Septic Shock’’ OR ‘’Shock, Toxic’’ OR ‘’Toxic Shock Syndrome’’ OR ‘’Toxic Shock Syndromes’’ OR ‘’Shock, Endotoxic’’ OR ‘’Endotoxin Shock’’ OR ‘’Endotoxin Shocks’’

Supplementary Table 2. In-progress clinical trials

Study	Record	URL
The Effect of Early Use of Methylene Blue on Hemodynamics in Septic Shock	NCT04970602	https://clinicaltrials.gov/show/NCT04970602
Methylene blue as vasopressor in septic shock	CTRI/2021/02/031514	https://trialsearch.who.int/Trial2.aspx?TrialID=CTRI/2021/02/031514
Effect of methylene blue on hemodynamic and metabolic response in septic shock patients	RBR-96584w4	https://doi.org/10.1097/MD.0000000000028599

Supplementary Table 3: Summary of the certainty of the evidence, a figure created using the GRADE.

Certainty assessment							№ of patients		Effect		Certainty	Importance
№ of studies	Study design	Risk of bias	Inconsistency	Indirectness	Imprecision	Other considerations	Methylene blue	Placebo	Relative (95% CI)	Absolute (95% CI)		
Mortality												
3	randomised trials	not serious	not serious	not serious	very serious[a]	none	24/70 (34.3%)	32/71 (45.1%)	**OR 0.62** (0.31 to 1.25)	**114 fewer per 1.000** (from 248 fewer to 56 more)	⊕⊕◯◯ Low	CRITICAL
Intensive care unit length of stay												
3	randomised trials	not serious	serious[b]	not serious	serious[c]	none	70	71	-	MD **1.58 days lower** (2.97 lower to 0.2 lower)	⊕⊕◯◯ Low	IMPORTANT
Time to vasopressor discontinuation												

Certainty assessment							№ of patients		Effect		Certainty	Importance
№ of studies	Study design	Risk of bias	Inconsistency	Indirectness	Imprecision	Other considerations	Methylene blue	Placebo	Relative (95% CI)	Absolute (95% CI)		
2	randomised trials	not serious	not serious	not serious	serious[c]	none	55	56	-	MD **31.49 hours lower** (46.02 lower to 16.96 lower)	⊕⊕⊕◯ Moderate	IMPORTANT
Days on mechanical ventilation												
3	randomised trials	not serious	serious[b]	not serious	serious[c]	none	70	71	-	MD **0.72 days lower** (1.26 lower to 0.17 lower)	⊕⊕◯◯ Low	IMPORTANT
Mean arterial pressure in 24 hours												
3	randomised trials	not serious	not serious	not serious	very serious[a]	none	70	71	-	MD **3.56 mmHg higher** (1.99 lower to 9.11 higher)	⊕⊕◯◯ Low	IMPORTANT
Methemoglobinemia (%)												
3	randomised trials	not serious	very serious[d]	not serious	very serious[a]	none	70	71	-	MD **0.85 % higher** (0.44 lower to 2.15 higher)	⊕◯◯◯ Very low	NOT IMPORTANT

CI: confidence interval; **MD:** mean difference; **OR:** odds ratio

Explanations

a. No optimal information size, large confidence interval (crossing central line)

b. there is variation in the effect estimate

c. No optimal information size

d. High heterogeneity statistically significant ($I^2 = 99\%$, $p < 0.00001$), no no IC95% overlap.
